# Results from a pre-post, uncontrolled pilot study of a mindfulness-based program for early elementary school teachers

**DOI:** 10.1186/s40814-020-00718-7

**Published:** 2020-11-16

**Authors:** Summer S. Braun, Robert W. Roeser, Andrew J. Mashburn

**Affiliations:** 1 University of Virginia, 405 Emmet Street South, PO Box 400281, Charlottesville, VA 22904 USA; 2 The Pennsylvania State University, 115 Health and Human Development, University Park, PA 16801 USA; 3 Portland State University, 317 Cramer Hall, 1721 SW Broadway, Portland, OR 97207 USA

**Keywords:** Elementary school, Elementary teachers, Teacher well-being, Mindfulness intervention, Professional development, Emotion regulation, Teaching practices

## Abstract

**Background:**

Mindfulness-based programs are a novel and promising approach for supporting teachers’ occupational health and well-being. Although rationales for mindfulness programs for teachers have been offered, the empirical research base evaluating approaches for educating teachers in mindfulness is still developing. This study reports the findings of a pilot study of a mindfulness-based program. This study is unique in that it is one of the only studies of the Mindfulness-Based Emotional Balance (MBEB) program to focus on early elementary teachers, to be implemented by a new instructor, and to recruit teachers via extrinsic motivators.

**Methods:**

A pre-post, uncontrolled pilot study of a 27.5-h mindfulness-based program for teachers was conducted with 21 pre-kindergarten–third-grade teachers from the Pacific Northwest of the USA. Program acceptability was assessed based on attendance and teacher reports of program benefits. Effect sizes for within-person changes (from pre- to post-program) in teachers’ skills and mindsets, well-being, occupational health, and teaching practices were calculated. Teachers also suggested improvements to the program.

**Results:**

With regard to program attendance and acceptability, teachers attended 87% of sessions, with 58% of teachers reporting a personal benefit and 58% of teachers reporting a professional benefit of the program. Effect sizes for changes in teachers’ skills and mindsets ranged from small to large, |*d*| = 0.30 to 0.83, and ranged from small to medium for changes in teachers’ well-being |*d*| = 0.07 to 0.48, occupational health |*d*| = 0.14 to 0.39, and teaching practices |*d*| = 0.15 to 0.48. Teachers suggested shortening the program and linking it more closely to their work in the classroom.

**Conclusions:**

This study suggests that the MBEB program may be beneficial to early elementary teachers, even when implemented by someone other than the program developer, and when provided with extrinsic motivation to participate (more closely mapping to a larger-scale trial of the program). Teachers’ suggestions regarding program length and structure are considered, along with useful avenues for future research on mindfulness-based programs for teachers.

## Key messages regarding feasibility

- *What uncertainties existed regarding feasibility?* The feasibility of the Mindfulness-Based Emotional Balance (MBEB) program when implemented for early elementary educators was unknown. It was also unclear how extrinsic motivation and implementation by someone other than the program developer might impact feasibility.
- *What are the key feasibility findings?* Teachers attended 87% of sessions; 58% of teachers reported personal and professional benefits of the program. Effect sizes on program outcomes ranged from small to large |*d*| = 0.07 to 0.83, and teachers offered suggestions for adapting the program.
- *What are the implications of the feasibility findings for the design of the main study?* Findings suggest that the program can be offered to a wide array of teachers, that those trained by the program developer can competently implement the program, and that compensating participants may not have deleterious impacts on feasibility or program outcomes.

## Introduction

Teachers’ occupational health and well-being are important for their overall quality of life, as well as for the well-being of their classes and students [7, 38]. Mindfulness training, focused on the cultivation of conscious, curious, nonjudgmental, and present-centered states of awareness [29], has emerged as a novel way to teach the cognitive and emotional skills theorized to support teachers’ occupational health and well-being [45]. Although several conceptual rationales for teaching mindfulness to teachers have been offered [24, 46], a recent meta-analysis of existing mindfulness-based programs for teachers concluded that the empirical research of these programs is still at its infancy [15]. The small body of existing research has shown promising effects for teachers [17, 19, 26, 45], with additional research necessary to demonstrate, with more rigorous study designs and objective measures, the conclusive power of these programs.

The present study contributes to this growing body of literature by presenting the results from a pre-post, uncontrolled pilot study of the Mindfulness-Based Emotional Balance (MBEB) program for early elementary teachers. This study aimed to (1) assess the program’s acceptability; (2) assess changes in teachers’ skills and mindsets (mindfulness skills, self-compassion, cognitive reappraisal, expressive suppression), well-being (mental health satisfaction, life satisfaction, anxiety symptoms, depressive symptoms), occupational health (personal accomplishment, emotional exhaustion, depersonalization, job stress), and classroom teaching practices (mindful teaching, emotional support, classroom organization) over the course of the program; and (3) explore teachers’ suggestions for improvements to the program.

## Logic model: theorized effects of a mindfulness-based program on teachers’ skills, well-being, occupational health, and teaching practices

**Fig. 1:**
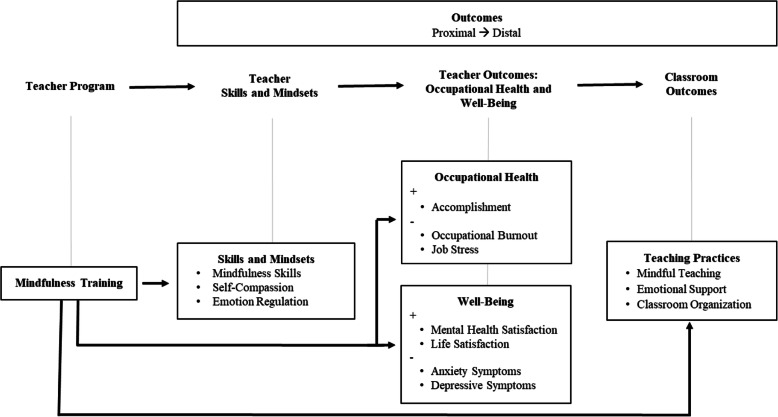
Logic model of the Mindfulness-Based Emotional Balance program for early elementary school teachers

The logic model for the MBEB program is depicted in Fig. 1. Mindfulness training is hypothesized to build teachers’ cognitive and emotional resources, which provide teachers with additional resources for coping with everyday challenges, thereby improving their well-being. Social-cognitive theories of job stress and coping posit that feelings of job stress emerge when an individual’s resources are insufficient to manage the demands of the situation [32]. By cultivating these cognitive and emotional skills through mindfulness training, teachers are theorized to feel more capable of meeting the demands of their profession, whereby improving occupational health. With more energy and less stress, teachers are hypothesized to invest resources in classroom interactions with students around learning (see also [46]).

### Relation to teachers’ skills and mindsets

Previous studies of mindfulness-based programs for teachers have shown them to be efficacious in fostering change in (i.e., learning of) mindfulness skills and a self-compassionate mindset [17, 25, 27, 45]. Mindfulness practice can also improve mindful emotion regulation by increasing awareness of somatic and emotional arousal, as well as the implementation of mindful coping strategies to address such arousal (e.g., pausing and breathing; see [23]). Existing studies suggest that teachers who have acquired mindfulness and self-compassion skills are more accepting of their own emotions, able to reappraise situations in less stressful ways, and are less likely to suppress emotional expression [15, 25, 53]. Emotion regulation skills are important because they are a central factor implicated in teachers’ levels of work-related stress [36].

Of particular interest in this study is teachers’ use of two emotion regulation strategies: cognitive reappraisal and suppression of emotional expression. Cognitive reappraisal involves a re-conceptualization of a potentially emotion-eliciting situation in a more positive or less emotional way. It is a healthy regulation strategy which is associated with higher levels of positive affect and lower levels of negative affect, depressive, and anxiety symptoms [21]. Expressive suppression is the modification of the behavioral expression of emotion and is a maladaptive strategy associated with exacerbated feelings of negative emotions, negative affect and depressive symptoms, low levels of positive affect, life satisfaction, and job satisfaction and even students’ less positive outlook and fewer prosocial behaviors [7, 21, 52].

### Relation to well-being

Well-being has been conceptualized as the presence of pleasant (e.g., life satisfaction) and the absence of unpleasant (e.g., depressive symptoms) emotional experiences [30]. Through mindfulness and mindful emotion regulation, mindfulness training is thought to improve teachers’ personal well-being (Chambers et al., [10]). For instance, because mindful individuals employ strategies like cognitive reappraisal to put challenging situations in perspective, they are less likely to feel overwhelmed, irritable, anxious, or defensive about current experiences. Existing studies have demonstrated reductions in teachers’ anxious and depressive symptoms, bad moods at home, and greater home satisfaction and more sleep over the course of their participation in mindfulness-based programs [12, 19, 45].

### Relation to occupational health

Teachers’ occupational health has been conceptualized as the presence of pleasant (e.g., job satisfaction) and absence of unpleasant (e.g., emotional exhaustion) emotional experiences in the work context [2, 30]. It is hypothesized that teachers apply mindfulness and emotion regulation skills in ways that positively impact their occupational health (e.g., job stress and satisfaction). Empirical support for these associations come from studies of the MBEB and Cultivating Awareness and Resilience in Education (CARE) for Teachers program with MBEB teachers reporting lower occupational burnout (specifically, less emotional exhaustion) and job stress, less frequent bad moods at work, and greater satisfaction at work than control teachers [12, 45], and CARE teachers reporting less psychological distress, a composite of unpleasant occupational and personal well-being indicators, and occupational burnout compared to control teachers [25, 27].

### Relation to teaching practices

Mindfulness skills are theorized to improve teachers’ practices in part through the adoption of an attitude of kindness towards oneself and others [14], which may itself allow teachers to directly engage in responsive, high-quality interactions with students. Mindfulness training may indirectly affect the quality of teachers’ interactions with students in the classroom by reducing feelings of emotional exhaustion and depersonalization, which increase teachers’ energy and capacity to empathize with students [45, 51]. Initial support for the association between mindfulness-based programs and teaching practices have been documented in CARE teachers engaging in more emotionally supportive interactions with students than control teachers at post-program [25].

## Mindfulness-Based Emotional Balance program

The current study focuses on the 8-week, 9-session version of the MBEB program, which met after school for a total of 27.5 h [13]. In comparison to the 8-week, 11-session, 36-h version of MBEB as reported in Roeser et al. [45], this version differs in the following ways: material originally covered in sessions 2 and 4 were covered in one session; material originally covered in sessions 7 and 8 were covered in one session; and the length of the retreats was shortened to 5 h each. The program was led by a trained clinical psychologist who was also an instructor of mindfulness and movement. This was the first time this instructor delivered this program, and the new instructor was coached and trained by the program developer, Margaret Cullen.

The topics covered in each session of MBEB are detailed in Additional file 1. Approximately 30% of the MBEB program is devoted to mindfulness-based emotion skills, 50% to mindfulness-based stress reduction, and 20% to mindfulness-based prosocial dispositions. The program aims to cultivate teachers’ abilities to direct and sustain attention intentionally and nonjudgmentally on present-moment experiences through a variety of teaching formats and guided home practices. Teachers are invited to apply the content to their lives during the following week and discuss their experiences with the practices and content during each session. For more detailed program information, see Benn, Akiva, Arel, & Roeser [6], Cullen & Pons [13], and Roeser et al. [45].

One important note about the MBEB program in relation to other mindfulness-based programs is that the MBEB program does not aim to scaffold and transfer program-related skills specifically to the classroom context, but rather invites teachers to explore and use these skills in any area of their lives that they wish. This could include their work context, but it need not (see Roeser, [47]). In only one case are teachers explicitly asked to apply the content to their classrooms—in a homework assignment to practice loving-kindness meditation for a student in their classroom that they struggle with. Besides this, the program offers a platform for teachers to learn the skills and apply them in their lives as they see fit, without specific directives to use mindfulness in any particular way or context. In this way, instructors aim to support the wisdom and autonomy of the teachers taking the program.

## Early elementary teachers

To date, the MBEB program has been tested on parents and teachers of elementary-high school students with special needs, and elementary and secondary teachers. The program has demonstrated preliminary positive effects on participants’ skills and mindsets, well-being, and teachers’ occupational health [6, 45]. The focus of this study was on pre-kindergarten to third-grade teachers who serve young students as they transition into formal schooling. Meeting the diverse needs of 30 young students who are socially, emotionally, behaviorally, and academically immature, for the entire day (i.e., these students do not switch teachers during the day), are conditions that do not exist in the same way for later elementary and secondary school teachers, and are major sources of stress for early elementary teachers [41, 43]. Thus, the present study contributes to the growing body of literature on the MBEB program by studying for the first time the effects of MBEB on early elementary school teachers. This study is distinct from other tests of MBEB in two additional ways: the current program was implemented by a new instructor, rather than the program developer, as was the case for previous studies of MBEB, and this study assessed program impacts when teachers may have been more extrinsically (e.g., school leadership encouragement and payment) than intrinsically motivated (e.g., suffering) to participate, which may more closely represent the teachers who would participate in the program should it be scaled up and disseminated more widely.

## Present study

Pilot studies are a useful step in the research process which help to determine the feasibility and acceptability of a program and to explore possible population-specific adaptations [3, 8, 55]. This pilot study of the MBEB program for early elementary school teachers aimed to address the following research questions:

RQ_1_: To what extent did teachers attend and report benefitting from the program?

RQ_2_: Did teachers’ skills and mindsets, well-being, occupational health, and teaching practices improve across the program?

RQ_3_: How could the program be adapted to better suit the needs of early elementary teachers?

We hypothesized that teachers would experience improvements in their skills and mindsets, well-being, occupational health, and teaching practices over the course of the program. Due to the novelty of implementing this program with early elementary teachers, the investigation into teachers’ acceptability and suggested adaptations were exploratory and meant to inform future iterations of the program.

## Method

### Design and procedure

The present study employed a pre-post, no-control group design [8, 16, 22, 39, 54]. Data come from two sequential implementations of the MBEB program for pre-kindergarten to third-grade teachers implemented in two sites in an urban city in the pacific northwestern USA: site 1 in the fall of 2013 and site 2 in the fall of 2014. The study was reviewed and approved by the human-subjects research review committee at Portland State University. Given the study’s primary motivation of assessing the feasibility of implementing the MBEB program under these novel conditions, a formal sample size calculation was not warranted, with the goal of recruiting as many teachers within the participating sites as possible [33]. Each site received the MBEB program for teachers and a social and emotional learning curriculum for students. The present study focuses on the MBEB program and changes in teachers’ experiences and teaching practices.

Data were collected at two time points: at the start of the school year (prior to the program) and at the end of the school year (after the program). Teachers completed an online survey containing questions regarding their demographics, skills and mindsets, well-being, and occupational health at each assessment. At each time point, teachers were videotaped in their classrooms on 3 days for 20 min. At post-program, teachers also answered questions regarding the MBEB program in an online survey.

### Participants

The study drew teachers from two elementary schools with linked preschool sites whose principals/leaders, with district support, were interested in introducing mindfulness and social-emotional learning to their teachers and students. Schools were designated as Title 1 and served primarily economically disadvantaged families and children. In recent years, the schools increasingly served immigrant families from Russia and Mexico, although all teachers were European-American. The teachers in this study were encouraged by their principal to participate but could opt in or out of the program. Participating teachers were paid for the time they devoted to the program. Thus, unlike other studies of MBEB in which all teachers were self-selected, the motivation for teachers to participate in this study included encouragement from school leadership and financial payment for time. Participating teachers provided their informed consent.

As teachers’ experiences at pre-program did not differ by site, data were collapsed across the two sites/years of the study. Teachers had a range of teaching experience (*M* = 12 years, *SD* = 8); were 86% female, 100% Caucasian; and 71% had a post-baccalaureate diploma or graduate degree. In terms of grade, 10% were pre-kindergarten teachers (*N* = 2), 14% kindergarten teachers (*N* = 3), 28% first-grade teachers (*N* = 6), 10% second-grade teachers (*N* = 2), and 38% third-grade teachers (*N* = 8).

### Measures

#### Attendance and acceptability

The number of teachers who enrolled in the study and participated at each time point was recorded. *Attendance* was taken at each program session. Teachers responded to three questions about the *acceptability* of the program: “How much would you say you’ve benefitted personally from this program?” and “How much would you say you’ve benefitted professionally from this program?” Responses were scored on a 1 to 5 scale (1 = *Not at all or very slightly*; 5 = *Benefitted a great amount*). Teachers were also asked “Would you recommend this program to your peers who are teachers?” Responses were *Yes*, *No*, and *Unsure*.

#### Teacher skills and mindsets

Teachers self-reported on their mindfulness skills, occupational self-compassion, and emotion regulation skills.

*Mindfulness skills* were measured using 38 items from the Five Factor Mindfulness Questionnaire [1], which assessed five components of teachers’ mindfulness skills: mindful observing, describing, active awareness, nonjudgement, and nonreactivity (*α*_pre_ = .92, *α*_post_ = .91). Items were scored on a 1 to 5 scale (1 = *Almost Never*; 5 = *Almost Always*). Items included “When I’m walking, I deliberately notice the sensations of my body moving” (observing), “I’m good at finding words to describe my feelings” (describing), “When I do things, my mind wanders off and I’m easily distracted” (active awareness), “I tell myself I shouldn’t be feeling the way I’m feeling” (nonjudgement), and “In difficult situations, I can pause without immediately reacting” (nonreactivity). Items were scored so higher values indicate greater mindful skills.

*Self-compassion* was measured using Roeser et al.’s [45] adaptation of Neff’s [37] self-compassion scale for teachers (*α*_pre_ = .85, *α*_post_ = .78). The scale assesses teachers’ extension to compassion to themselves in the context of their teacher role. Items were scored on a 1 to 5 scale (1 = *Not at all True*; 5 = *Very True*; e.g., “As a teacher, I’m tolerant of my own flaws and inadequacies”).

*Cognitive reappraisal* was measured using six items from the Emotion Regulation Questionnaire ([20]; *α*_pre_ = .88, *α*_post_ = .86). Items were scored on a 1 to 5 scale (1 = *Strongly Disagree*; 5 = *Strongly Agree*; e.g., “I control my emotions by changing the way I think about the situation I’m in”).

*Expressive suppression* was measured using four items from the Emotion Regulation Questionnaire ([20]; *α*_pre_ = .67, *α*_post_ = .80). Items were scored on a 1 to 5 scale (1 = *Strongly Disagree*; 5 = *Strongly Agree*; e.g., “I control my emotions by not expressing them”).

#### Well-being

Was measured using indicators of life and emotional satisfaction, as well as teachers’ reports of anxious and depressive symptoms.

*Mental health satisfaction* was assessed using a single item, “Overall, how satisfied are you with your emotional/mental health these days?” Items were scored on 1 to 4 scale (1 = *Not at all Satisfied*; 4 = *Very Satisfied*).

*Life satisfaction* was assessed using a single item. “Taking all things together, how satisfied are you with your life as a whole these days?” Items were scored on 1 to 4 scale (1 = *Not at all Satisfied*; 4 = *Very Satisfied*)*.*

*Anxiety symptoms* were measured using six items from the State/Trait Anxiety Inventory ([50]; *α*_pre_ = .78, *α*_post_ = .90). Items were scored on a 1 to 4 scale (1 = *Not at all*; 4 = *Very Much*; e.g., “I feel nervous”).

*Depressive symptoms* were measured using 12 items from the Beck Depression Inventory ([5]; *α*_pre_ = .69, *α*_post_ = .81). Teachers were prompted about a topic and selected the option (1 to 4) that most accurately described their current state. For example, one item prompted about sleep habits; options included the following: I can sleep as well as usual (1), I don’t sleep as well as I used to (2), I wake up 1-2 hours earlier than usual and find it hard to get back to sleep (3), and I wake up several hours earlier than usual and cannot get back to sleep (4). Higher values indicate greater depressive symptoms.

#### Occupational health

Was measured as teachers’ feelings of personal accomplishment, emotional exhaustion, depersonalization, and job stress.

*Personal accomplishment* was measured using seven items from the Maslach Burnout Inventory ([34]; *α*_pre_ = .79, *α*_post_ = .89). Items were scored on a 1 to 7 scale (1 = *Never*; 7 = *Every Day*; e.g., “How often do you easily understand how your students feel about things?”); higher values indicate stronger feelings of burnout.

*Emotional exhaustion* was measured using six items from the Maslach Burnout Inventory ([34]; *α*_pre_ = .92, *α*_post_ = .91). Items were scored on a 1 to 7 scale (1 = *Never*; 7 = *Every Day*; e.g., “How often do you feel emotionally drained from your work?”); higher values indicate stronger feelings of burnout.

*Depersonalization* was measured using five items from the Maslach Burnout Inventory ([34]; *α*_pre_ = .87, *α*_post_ = .76). Items were scored on a 1 to 7 scale (1 = *Never*; 7 = *Every Day*; e.g., “How often do you feel you treat some student as if they were impersonal objects?”); higher values indicate stronger feelings of burnout.

*Job stress* was measured using nine items ([45]; *α*_pre_ = .72, *α*_post_ = .71). Items were scored on a 1 to 5 scale (1 = *Strongly Disagree*; 5 = *Strongly Agree*; e.g., “I feel dealing with student discipline problems puts a lot of stress on me”).

#### Teaching practices

Were measured using teachers’ reports of their mindfulness while teaching, observer-rated emotionally supportive interactions with students, and classroom organization.

*Mindful teaching* was measured using 20 items from the Mindfulness in Teaching Scale ([18]; *α*_pre_ = .85, *α*_post_ = .76). Items were scored on a 1 to 5 scale (1 = *Almost Never*; 5 = *Almost Always*; e.g., “I am aware of how my moods affect the way I treat my students”).

*Emotional support and classroom organization* were assessed using the Classroom Assessment Scoring System (CLASS [40];). At each time point, teachers were videotaped three times for 20 min. Three certified CLASS observers watched the videos while taking notes about the quality of teacher-student interactions and assigned ratings about the quality of interactions for each dimension (1 = *Low*, 7 = *High*): positive climate, negative climate, sensitivity, regard for student perspective, behavior management, productivity, and instructional learning formats. Approximately 35% of these videos were independently rated by two observers, inter-rater reliability > .85. Consistent with our logic model [46], scales were created for emotional support and classroom organization. *Emotional support* reflects teachers’ positive and negative classroom climate, their sensitivity to students, and regard for student perspective, including supporting student autonomy, ideas, and peer interactions. *Classroom organization* reflects teachers’ clear, proactive management of student behavior, productive use of time, and employment of a variety of learning formats that encourage student engagement. Observer scores for each time point were averaged such that each teacher received a score for observed emotional support and classroom organization at pre-program and at post-program.

#### Suggested adaptations

Teachers were asked two questions about the program. The first, “How do you feel about the length of the program?” was scored on a 1 to 5 scale (1 = *Much too long*; 5 = *Much too short*). The second was an open-ended question, “What suggestions, if any, do you have for the instructor for making this program better in the future?”

### Data analysis

#### Attendance and acceptability

The number of participating teachers was recorded at each timepoint in the study. The average proportion of sessions attended was calculated, along with the proportion of teachers who missed one or no sessions. The number of absences was also determined. Acceptability was calculated using the proportion of teachers who reported that they “benefitted a moderate amount” or more (scores 3–5). The proportion of teachers who would recommend the program to fellow teachers was also determined.

#### Teacher and teaching outcomes

Descriptive statistics are provided in Table 1; correlations among variables of interest at pre-program are displayed in Table 2. Effect sizes between pre-program and post-program were calculated in R using Cohen’s *d* and correcting for dependence between means ([54]; Fig. 3), with effect sizes of *d* = 0.2 considered small, *d* = 0.5 considered medium, and *d* = 0.8 considered large [42, 49]. Confidence intervals for these effect sizes are displayed as error bars in Fig. 3.

**Table 1:** Descriptive statistics for teachers’ skills and mindsets, well-being, occupational health, and teaching practices at pre- and post-program

	Pre-Program					Post-Program				
	*N*	Mean	Median	*SD*	IQR	*N*	Mean	Median	*SD*	IQR
Skills and Mindsets										
Mindfulness Skills	21	3.17	3.26	0.47	0.62	21	3.47	3.53	0.42	0.78
Self-Compassion	21	2.97	3.00	0.70	0.93	21	3.24	3.29	0.59	1.06
Cognitive Reappraisal	21	3.59	4.00	0.68	1.00	21	3.96	4.00	0.46	0.25
Expressive Suppression	21	2.55	2.50	0.66	1.13	21	2.38	2.25	0.68	0.88
Well-Being										
Mental Health Satisfaction	21	2.67	3.00	0.66	1.00	21	3.00	3.00	0.71	0.00
Life Satisfaction	20	3.25	3.00	0.55	1.00	21	3.14	3.00	0.57	0.50
Anxiety Symptoms	21	2.33	2.33	0.57	0.92	21	2.02	2.00	0.67	1.08
Depressive Symptoms	21	1.34	1.25	0.23	0.27	21	1.32	1.25	0.30	0.46
Occupational Health										
Personal Accomplishment	21	6.13	6.13	0.66	0.88	21	6.24	6.50	0.83	0.95
Emotional Exhaustion	21	4.57	4.50	1.09	1.76	21	4.13	4.00	1.28	1.28
Depersonalization	21	1.97	1.60	1.24	1.03	21	1.84	1.60	0.97	1.40
Job Stress	21	3.39	3.44	0.51	0.72	21	3.22	3.22	0.48	0.78
Teaching Practices										
Mindful Teaching	21	3.61	3.60	0.41	0.65	21	3.81	3.75	0.32	0.50
Emotional Support	21	5.50	5.75	0.55	0.88	19	5.65	5.75	0.53	0.74
Classroom Organization	21	5.46	5.42	0.73	0.90	19	5.53	5.67	0.43	0.74

*Note. N* = Participants with data; *SD* = Standard deviation

**Table 2:** Correlations among teachers’ skills and mindsets, well-being, occupational health, and teaching practices at pre-program assessment

			Skills & Mindsets				Well-Being				Occupational Health				Teaching Practices		
			1	2	3	4	5	6	7	8	9	10	11	12	13	14	15
Skills & Mindsets	1	Mindfulness Skills	-														
	2	Self-Compassion	0.52	-													
	3	Cognitive Reappraisal	0.53	0.53	-												
	4	Expressive Suppression	-0.58	-0.08	-0.08	-											
Well-Being	5	Mental Health Satisfaction	0.40	0.12	0.54	-0.05	-										
	6	Life Satisfaction	0.03	0.33	0.26	0.13	0.22	-									
	7	Anxiety Symptoms	-0.27	-0.45	-0.56	-0.22	-0.62	-0.65	-								
	8	Depressive Symptoms	-0.25	-0.30	-0.59	-0.06	-0.39	-0.36	0.60	-							
Occupational Health	9	Personal Accomplishment	0.64	0.44	0.64	-0.08	0.40	0.22	-0.47	-0.38	-						
	10	Emotional Exhaustion	0.03	0.27	-0.34	0.10	-0.17	-0.15	0.15	0.46	-0.12	-					
	11	Depersonalization	0.04	0.16	0.02	0.19	0.04	0.25	-0.17	0.15	0.07	0.56	-				
	12	Job Stress	-0.40	-0.15	-0.56	0.22	-0.20	-0.31	0.33	0.71	-0.44	0.52	-0.09	-			
Teaching Practices	13	Mindful Teaching	0.71	0.58	0.45	-0.54	0.07	-0.01	-0.13	-0.25	0.59	-0.18	-0.45	-0.29	-		
	14	Emotional Support	0.07	-0.01	-0.08	-0.38	0.22	0.03	0.05	0.01	0.07	-0.31	-0.47	0.04	0.33	-	
	15	Classroom Organization	-0.01	0.02	-0.05	-0.25	0.13	-0.12	0.05	-0.18	0.02	-0.28	-0.58	0.01	0.29	0.85	-

*Note. p* < .05 when |*r*| > .38

#### Suggested adaptations

The average response regarding program length was calculated. Teachers’ free responses regarding their suggestions were coded using qualitative content analysis [35]. Each response could contain multiple themes. The frequency of each theme was calculated.

## Results

### Attendance and acceptability

**Fig. 2:**
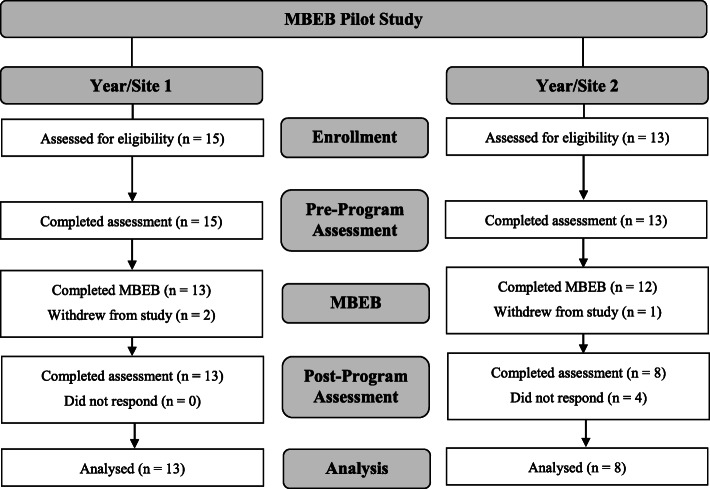
Participants at each stage of the research process

During the first year of the project, 15 teachers from site 1 enrolled in the program; two teachers withdrew after the initial wave of data collection, but prior to program participation (Fig. 2). During the second year of the project, 13 teachers from site 2 enrolled in the program; one teacher withdrew after two program sessions, and four did not complete data collection. In sum, 21 teachers participated in the study and completed assessments at pre-program and post-program: 13 teachers from site/year 1 and 8 teachers from site/year 2.

Attendance for the 21 participating teachers was generally high, with 76% of participating teachers missing one or no sessions. On average, teachers attended 87% of sessions. Of the missed sessions, 31% were planned absences, 17% were sickness-related, and 52% were unannounced.

Teachers reported on the personal benefit of the program, with 58% of teachers reporting that they benefitted “a moderate amount” or higher. Teachers also reported on the professional benefit of the program, with 58% of teachers reporting that they benefitted “a moderate amount” or higher. Half of the teachers reported that they would recommend the program to fellow teachers, 33% were unsure, and 16% reported that they would not recommend the program.

### Teacher and teaching outcomes

**Fig. 3:**
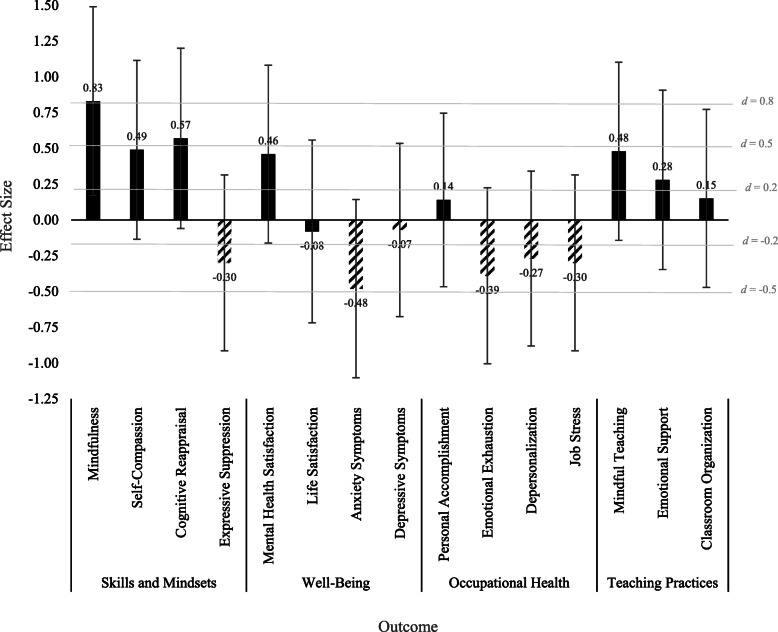
Effect sizes (Cohen’s *d* with confidence intervals) for within-person changes in teachers’ skills and mindsets, well-being, occupational health, and teaching practices. Solid bars indicate outcomes hypothesized to increase over time, and striped bars indicate outcomes hypothesized to decrease over time

Effect sizes for within-person change in each outcome between pre-program and post-program are displayed in Fig. 3. Effect sizes for changes in teachers’ skills and mindsets ranged from small to large |*d*| = 0.30 to 0.83 and ranged from small to medium for changes in teachers’ well-being |*d*| = 0.07 to 0.48, occupational health |*d*| = 0.14 to 0.39, and teaching practices |*d*| = 0.15 to 0.48.

### Suggested adaptations

Regarding program length, the average response was 2.33, indicating that most teachers reported that the program was “a little too long.” With regard to teachers’ suggestions, of the 12 teachers who responded to the post-program survey, half did not provide a response to this question. Responses for the six teachers who responded were coded thematically using qualitative content analysis. Three codes emerged: positive comments or responses suggesting no changes; suggestions to streamline the program either through shortening the sessions or limiting repetition of content; and suggestions for explicit instruction regarding the application of program content to teaching. Of the six respondents, three had positive comments or suggested no changes. Two teachers suggested streamlining the program, and two suggested explicit application of program content to teaching.

## Discussion

Results from this pilot study demonstrate that the MBEB program can be implemented with, and have positive effects for, early elementary school teachers. This study adds three new directions to the existing research on the feasibility and success of the MBEB program: (1) the application of this program to early elementary teachers, (2) a test of program effects upon implementation by a new instructor, and (3) a test of program impacts when teachers were extrinsically motivated (e.g., school leadership encouragement and payment vs. intrinsically motivated [e.g., suffering]) to participate. Under these novel conditions for program implementation, teachers reported improvements over the course of the program in all domains assessed. These findings suggest that the program may continue to have positive benefits when it is offered to a wider array of teachers, that those trained by the program developer can competently implement the program, and that compensating participants may not have deleterious impacts on feasibility or program outcomes. These findings have important implications for the scale-up and dissemination of the MBEB program, together suggesting that the MBEB program holds promise for school-wide implementation. This study adds to the growing body of literature on mindfulness-based programs for teachers and the promise of such programming for promoting positive change for teachers [15, 17, 25, 45].

Teachers’ feedback suggested two specific directions in which to adapt the program. First, analyses of teachers’ free responses suggested that some teachers wanted more scaffolding with regard to transferring mindfulness skills to the classroom, despite the program being presented as non-instrumental. This feedback suggests that the program may not have met some teachers “where they were” in terms of their wanting to work on mindfulness primarily for classroom change. Balancing the capacity of the program to meet teachers’ personal and professional needs will be useful in developing future iterations of the program. For example, creating time at the end of each session for tying the content to everyday challenges and dilemmas of teaching practice may better suit the needs of certain teachers *and* result in greater improvements for all teachers’ occupational health and teaching practices.

Teachers’ feedback also suggested that the program was a bit too long, which brings to light the complex interplay between dosage, duration, and culturally adapted programming, which the field of mindfulness-based programs has yet to fully explore. A key challenge of prevention science is balancing the development of evidence-based prevention programs that can be implemented with fidelity, while also catering to the needs and wants of those the program aims to support [4, 9]. For example, a study of adaptations of an empirically supported family-based intervention found that shorting the program increased retention, but also reduced positive outcomes [31]. In the future, it will be important to investigate how this program may be shortened to best fit the busy schedules of early elementary teachers, without jeopardizing the integrity of the program’s content.

A strength of this study was its assessment of both pleasant and unpleasant feelings. With most research on teachers' occupational health and well-being focused on the reduction of unpleasant emotions like stress and burnout (e.g., [11, 48, 51]), this pilot study demonstrated that the MBEB program has the potential not only to alleviate unpleasant feelings, but also promote positive feelings, such as teachers’ satisfaction with their mental health. This is consistent with the idea that mindfulness and compassion training may reduce suffering *and* cultivate flourishing [44].

This pilot study has several limitations, including a reliance primarily on teacher self-report measures, and its focus on a small, racially homogenous sample. In addition, the processes through which these effects were inferred were not directly tested. This is a useful area of research that would contribute to our understanding of how effects are achieved and could inform program refinements that target such pathways. There is much still to be learned about the MBEB program for teachers and about the feasibility of mindfulness-based programs more generally. This pilot study brought to light several considerations that warrant further exploration, including the best approach to teaching mindfulness skills (e.g., through explicit application to teachers’ lives in the classroom), finding the “correct” dosage, and whether such programs are suitable for a diverse group of teachers.

## Conclusion

The present study addresses calls to understand how teachers’ social and emotional skills, well-being, occupational health, and teaching practices may be improved through novel means like mindfulness training (e.g., [28]). Results from the present study add to the emerging body of research suggesting that mindfulness-based programs can positively impact teachers and their classroom practice [15]. Pilot research, such as this study, is useful in elucidating challenges and unanswered questions—perhaps especially in a small and growing field like the research on teacher mindfulness programs. They can also help to find practical solutions to complex implementation issues on the way to more rigorous and large-scale testing of mindfulness-based programs (e.g., [3, 8]). The current study raises key issues to explore in future research. We look forward to new scientific insights on these key implementation issues as the field of mindfulness in education advances.

## Supplementary Information

**Additional file 1.** MBEB Program Sessions and Topics.

## Data Availability

The dataset analyzed in the current study is available from the corresponding author upon reasonable request.
