# Supplementary material for: Results from a pre-post, uncontrolled pilot study of a mindfulness-based program for early elementary school teachers
Source: Pilot Feasibility Stud. 2020 Nov 16;6:178. doi: 10.1186/s40814-020-00718-7 (PMC7667758; doi:10.1186/s40814-020-00718-7)
Supplement: Supplementary file 1 — Additional file 1. MBEB Program Sessions and Topics. [file 40814_2020_718_MOESM1_ESM.docx]

Appendix

*MBEB Program Sessions and Topics*

| Session | Topics |
| --- | --- |
|  |  |
| 1 | Introduction and Overview of Mindfulness Program |
| 2 | Mindfulness, Stress, Perception, and Emotion (Retreat 1) |
| 3 | Mindfulness, Stress, and Reacting vs. Responding |
| 4 | Mindfulness and Forgiveness |
| 5 | Mindfulness, Anger, and Kindness |
| 6 | Mindfulness, Fear, and Compassion |
| 7 | Mindfulness and Working with Conflict |
| 8 | Silent Day (Retreat 2) |
| 9 | Beginnings and Endings |
